# Convection heat mass transfer and MHD flow over a vertical plate with chemical reaction, arbitrary shear stress and exponential heating

**DOI:** 10.1038/s41598-021-81615-8

**Published:** 2021-02-19

**Authors:** Sehra, Sami Ul Haq, Syed Inayat Ali Shah, Kottakkaran Sooppy Nisar, Saeed Ullah Jan, Ilyas Khan

**Affiliations:** 1 Department of Mathematics, Islamia College Peshawar, Peshawar, 25000 Khyber Pakhtunkhwa Pakistan; 2 Department of Mathematics, College of Arts and Science at Wadi Aldawaser, Prince Sattam Bin Abdulaziz University, Alkharj, 11991 Kingdom of Saudi Arabia; 3 Department of Mathematics, College of Science Al-Zulfi, Majmaah University, Al-Majmaah, 11952 Kingdom of Saudi Arabia

**Keywords:** Engineering, Physics

## Abstract

The present research article is directed to study the heat and mass transference analysis of an incompressible Newtonian viscous fluid. The unsteady MHD natural convection flow over an infinite vertical plate with time dependent arbitrary shear stresses has been investigated. In heat and mass transfer analysis the chemical molecular diffusivity effects have been studied. Moreover, the infinite vertical plate is subjected to the phenomenon of exponential heating. For this study, we formulated the problem into three governing equations along with their corresponding initial and boundary conditions. The Laplace transform method has been used to gain the exact analytical solutions to the problem. Special cases of the obtained solutions are investigated. It is noticed that some well-known results from the published literature are achieved from these special cases. Finally, different physical parameters’ responses are investigated graphically through Mathcad software.

## Introduction

In daily life applications convection of heat-mass transfer analysis play a very important role. In the last few years natural convection flow theory highly developed and became the most rapidly established research field. A lot of phenomena’s relatable to convection flow over infinite vertical plate are studied in literature. In practical life heat and mass transfer procedure appears too much, such as chemical reaction evaporation, and also in condensation. Heat and mass transfer have also industrialized applications like the buoyancy effects of diffusion of chemical reactions and thermal diffusion; this is generated by thermal conduction and mass transfer analysis. Many researchers have been working in this field and eradicate many physical problems related to heat and mass transfer and investigated that problems analytically as well as graphically. The free convection unsteady flow of incompressible viscous fluid over a perpendicular plate together with ramped wall temperature is analyzed, and obtained the exact analytical solution to the problem by using the Laplace transform method^1^.The effects of heat and mass transfer over a movable vertical plate with ramped wall temperature is analyzed and obtained the exact analytical solution of the problem^2^. The channel flow of unsteady incompressible viscous fluid together with ramped wall temperature at single boundary, and obtained the exact solution by using the Laplace transform method^3^. A mathematical model investigated that the free convection isothermal diffusivity effect on unsteady viscous flow formed by Narahari et al.^4^. Rubbab et al. analyzed the free convection flow of viscous fluid that is closed to a perpendicular plate with arbitrary time dependent shear stresses^5^. Natural convection heat transfer phenomena of viscous incompressible fluid through permeable medium with magneto hydrodynamic flow analyzed^6^. In the last few years magneto hydrodynamic phenomenon in viscous fluid is an attractive research field for the scientists and engineers. These fields become very interesting for researchers caused by dynamic nature of the flow effects of magneto hydrodynamic and too many applications in industries as well as engineering problems. The magneto hydrodynamic could be found in many apparatus^7–9^. The MHD influences can be also used in plasma confinement, Astrophysical phenomena, liquid distortion into a metal, the nuclear reactors cooling, centrifugal pumps, humans body blood circulation, the medical study of breathing, electromagnetic casting, and numerous other physical and natural problems. The idea of MHD was firstly presented in 1942 by Hannes Alfven. He was also nominated for noble prize for his numerous services provided in the field of physical science. After a while this concept has been given a new idea to the researchers to study the velocity of fluid due to the magnetic field. Several researchers have taken the MHD influences in the fluid with the effects of the porosity^10–12^. Ferdows et al.^13^ analyzed mixed convection magneto hydro dynamic flow of Nano-fluids passes through a permeable mechanism subjected to exponentially extendable surface^14^. Free (natural) convection MHD flow of Nano-fluids via a permeable medium analyzed numerically^15^. Numerical approach to a mixed convection MHD flow of Nano fluids upon a porous medium investigated^16^. The analysis of MHD flow of Williamson with conduction radiation heat transfer with thermal diffusivity studied^17^. The chemical reaction mostly consist of a large number of well-known reactions such as exothermal reaction and isothermal reaction, these properties found in many industrial activities^18^. Many researchers worked on chemical reaction, and analyzed the chemical reaction phenomena, like effects of enzymatic reactions on thermic conduction and the mass transferring effects in a boundary surface subjected to many initial and boundary conditions^19–23^. Saeed et al. analyzed the thermal conduction and mass transference analysis in the existence of enzymatic reaction of free convection with the wall slip boundary condition^24^. There are two types of boundary conditions in the fluid flow phenomena, if the first layer of the fluid momentum which touches the surface of the plate is uniform to the velocity of that plate or boundary is called no-slip boundary condition and if that velocity not equal to boundary velocity is called slip boundary condition. Like in capillary action no-slip boundary conditions doesn’t applicable^25^. While Navier presented some limitations in his earlier work^26^. The slip boundary condition effect has too much applications for example Nano-channels or micro-channels. In the fluid problems slip condition has very important role in industries and chemical sciences. The steady flow together with magneto hydrodynamic passing through a channel with slip boundary condition studied by Makinde et al.^27^. The goal of the recent research is to analyze the slip wall influences, enzymatic reaction i.e. chemical reaction, and thermic exhaustion on incompressible viscous unsteady natural convection flow together with magneto hydrodynamic over a perpendicular plate with arbitrary shear stresses and exponential heating. The exact analytical solution for the dimensionless equations like temperature velocity and concentration equation is gained by applying the Laplace transformation method, for graphical representation Mathcad software, with the help of different physical parameters the exact analytical solution represented graphically.

## Mathematical formulation

**Figure 1:**
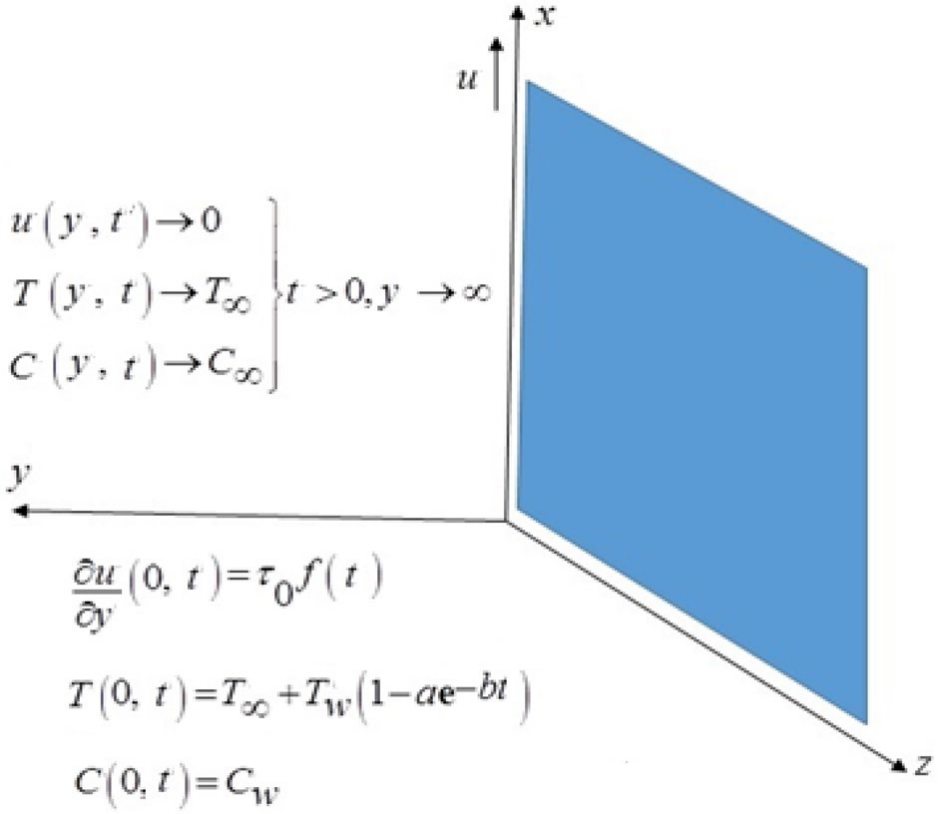
Physical model of the problem.

Considering the governing model equations in the dimensionless form with (initial-boundary) conditions subjected to the problem of free convection fluid flow of viscous fluid and with the property of incompressibility which passes through a perpendicular infinite plate and exponentially heated with arbitrary shear stresses applies to the fluid. Initially the fluid is at standstill mode for time $t = 0,$ the temperature is $T_{\infty }$ and concentration is $C_{\infty }$. As time begin to start at $t^{ + }$ the temperature and mass can be changed with the equations $T = T_{w} (1 - ae^{ - bt} ) + T_{\infty }$ and $C_{w}$ at respectively. For such a flow, the constraint of incompressibility is identically satisfied. Now by usual Boussinesq’s approximation^28,29^, the unsteady flow is governed by the following set of partial differential equations. The schematic diagram used in fluid flow problem is represented geometrically by Fig. 1.

1 $$\frac{\partial u(y,t)}{{\partial t}} = \nu \frac{{\partial^{2} u(y,t)}}{{\partial y^{2} }} - \frac{{\sigma B_{0}^{2} }}{\rho }u(y,t) + g\beta \left( {T(y,t) - T_{\infty } } \right) + g\gamma \left( {C(y,t) - C_{\infty } } \right),$$

2 $$\rho c_{p} \frac{\partial T(y,t)}{{\partial t}} = k\frac{{\partial^{2} T(y,t)}}{{\partial y^{2} }} - Q(T(y,t) - T_{\infty } ),$$

3 $$\frac{\partial C(y,t)}{{\partial t}} = D\frac{{\partial^{2} C(y,t)}}{{\partial y^{2} }} - K\left( {C(y,t) - C_{\infty } } \right).$$

The suitable (initial-boundary) conditions are:

4 $$u(y,t) = 0,\,\,T(y,t) = T_{\infty } ,\,\,C(y,t) = C_{\infty } ,\,\,t = 0, \, y \ge 0,$$

5 $$\frac{\partial u(y,t)}{{\partial y}} = \tau_{0} f(t),T(y,t) = T_{\infty } + T_{w} (1 - ae^{ - bt} ),C(y,t) = C_{w} ,\,y = 0\, \, a,\,b \ge 0, \, \,t > 0,$$

6 $$u(y,t) \to 0,\,T(y,t) \to T_{\infty } ,\,C(y,t) \to C_{\infty } \,{\text{as}}\,y \to \infty .$$

### Dimensionless variables

$$\begin{aligned} & y^{ * } = \frac{{U_{0} }}{\nu }y,\,\,\,u^{ * } = \frac{1}{{U_{0} }}u,\,\,\,t^{ * } = \frac{{U_{0}^{2} }}{\nu }t,\,\,\,T^{ * } = \frac{1}{{T_{w} }}\left( {T - T_{\infty } } \right),\,\,\,C^{ * } = \frac{1}{{C_{w} - C_{\infty } }}\left( {C - C_{\infty } } \right),\, \\ & {\text{Gr}} = \frac{{g \beta \nu T_{w} }}{{U_{0}^{3} }},M = \frac{{\sigma B_{0}^{2} \nu }}{{\rho U_{0}^{2} }},{\text{Gm}} = \frac{{g \gamma \nu (C_{w} - C_{\infty } )}}{{U_{0}^{3} }},{\text{Pr}} = \frac{{\rho c_{p} \nu }}{k}, \\ & \eta_{1} = \frac{Q\nu }{{\rho c_{p} U_{0}^{2} }},\eta_{2} = \frac{k\nu }{{U_{0}^{2} }},{\text{Sc}} = \frac{\nu }{D},b^{ * } = \frac{b\nu }{{U_{0}^{2} }},f^{*} (t^{*} ) = \frac{{\tau_{0} \nu }}{{U_{0}^{2} }}f\left( {\frac{\nu }{{U_{0}^{2} }}t^{*} } \right). \\ \end{aligned}$$

Adopting the overhead non-dimensional parameters in Eqs. (1)–(6), the dimensionless governing equations will have the simplest appearance as under as,

7 $$\frac{{\partial u^{*} }}{{\partial t^{*} }} = \frac{{\partial ^{2} u^{*} }}{{\partial y^{{*^{2} }} }} - Mu^{*} + {\text{Gr}}T^{*} + {\text{Gm}}C^{*} ,$$

8 $$\frac{{\partial T^{*} }}{{\partial t^{*} }} = \frac{1}{{{\text{Pr}}}}\frac{{\partial ^{2} T^{*} }}{{\partial y^{{*^{2} }} }} - \eta _{1} T^{*} ,$$

9 $$\frac{{\partial C^{*} }}{{\partial t^{*} }} = \frac{1}{{{\text{Sc}}}}\frac{{\partial ^{2} C^{*} }}{{\partial y^{{*^{2} }} }} - \eta _{2} C^{*} .$$

With non-dimensional (initial-boundary) conditions

10 $$u^{*} (y,t) = 0,\,\,T^{*} (y,t) = 0,\,\,C^{*} (y,t) = 0,\,\,t = 0, \, y \ge 0,$$

11 $$\frac{{\partial u^{*} }}{{\partial y^{*} }} = f^{*} \left( {t^{*} } \right),{\mkern 1mu} T^{*} (y,t) = (1 - ae^{{ - b^{*} t^{*}}} ),{\mkern 1mu} C^{*} (y,t) = 1,{\mkern 1mu} {\mkern 1mu} a,{\mkern 1mu} b \ge 0,{\mkern 1mu} t > 0,y = 0,$$

12 $$u^{*} (y,t) \to 0,\,T^{*} (y,t) \to 0,\,C^{*} (y,t) \to 0\,{\text{as}}\,y \to \infty .$$

## Analytical Laplace transformation solution

Now adopting the Laplace transformation to (7–9) equations with the (initial-boundary) conditions from (10–12). First, we find the concentration and temperature equations solutions because velocity equation is dependent on the (temperature-concentration) equations after that we will find solution for the velocity equation by applying Laplace transformation method. For the sake of convenience the * notation is omitted.

### Solution for concentration equation

$$\frac{{\partial^{2} \overline{C} }}{{\partial y^{2} }} - Sc\left( {s + \eta_{2} } \right)\overline{C} = 0.$$

With $$C(y,0) = 0,C(0,s) = \frac{1}{s}$$

13 $$\overline{C} = \frac{1}{s}\exp \left( { - y\sqrt {Sc\left( {s + \eta_{2} } \right)} } \right).$$

The exact analytical solution obtained by utilizing the Laplace inverse transform of the Eq. (13), we have the analytical solution for concentration profile which is given as under

$$C(y,t) = \frac{1}{2}\left[ {\text{erfc}\left( {\frac{y}{2}\sqrt {\frac{{Sc}}{t}} + \sqrt {\eta _{2} t} } \right)\exp \left( {y\sqrt {\eta _{2} Sc} } \right) + \text{erfc}\left( {\frac{y}{2}\sqrt {\frac{{Sc}}{t}} - \sqrt {\eta _{2} t} } \right)\exp \left( { - y\sqrt {\eta _{2} Sc} } \right)} \right].$$

### Solution for temperature equation

$$\frac{{\partial^{2} \overline{T} }}{{\partial y^{2} }} - {\text{Pr}}\left( {s + \eta_{1} } \right)\overline{T} = 0.$$

With $$\overline{T} (y,0) = 0, \, \overline{T} (0,s) = \frac{1}{s} - \frac{a}{s + b}$$

14 $$\overline{T} = \left( {\frac{1}{s} - \frac{a}{s + b}} \right)\exp \left( { - y\sqrt {{\text{Pr}}\left( {s + \eta_{1} } \right)} } \right).$$

The exact analytical solution obtained by applying the Laplace inverse transform of the Eq. (14), we have the analytical solution for temperature profile which is given as under.

$$\begin{aligned} T(y,t) & = \frac{1}{2}\left[ {\text{erfc}\left( {\frac{y}{2}\sqrt {\frac{\Pr }{t}} + \sqrt {\eta_{1} t} } \right)\exp \left( {y\sqrt {\eta_{1} \Pr } } \right) + \text{erfc}\left( {\frac{y}{2}\sqrt {\frac{\Pr }{t}} - \sqrt {\eta_{1} t} } \right)\exp \left( {y\sqrt {\eta_{1} \Pr } } \right)} \right] \\ & \quad - \frac{1}{2}\exp \left( {\eta_{1} t + \sqrt {\Pr } } \right)\left[ {\text{erfc}\left( {\frac{y}{2\sqrt t } - \sqrt {(\eta_{1} - b)t} } \right)\exp \left( { - y\sqrt {(\eta_{1} - b)} } \right) + \,\text{erfc}\left( {\frac{y}{2\sqrt t } + \sqrt {(\eta_{1} - b)t} } \right)\exp \left( {y\sqrt {(\eta_{1} - b)} } \right)} \right]. \\ \end{aligned}$$

### Solution for velocity equation

15 $$\frac{{\partial^{2} \overline{u} }}{{\partial y^{2} }} - \left( {s + M} \right)\overline{u} = - Gr\left( {\frac{1}{s} - \frac{a}{s + b}} \right)\exp \left( { - y\sqrt {\Pr \left( {s + \eta_{1} } \right)} } \right) - Gm\left( \frac{1}{s} \right)\exp \left( {y\sqrt {{\text{Sc}}\left( {s + \eta_{2} } \right)} } \right).$$

With $$\overline{u} (y,0) = 0\& \overline{u} (0,s) = f(s)$$.

Clearly Eq. (15) is non-homogenous so its solution will be in the form of

$$\overline{u} = \overline{u}_{c} + \overline{u}_{p}$$

$$\begin{aligned} \overline{u} (y,s) & = \frac{ - f(s)}{{\sqrt {\left( {s + M} \right)} }}{\text{exp}}\left( { - y\sqrt {\left( {s + M} \right)} } \right) - B_{1} \frac{{\sqrt {\Pr \left( {s + \eta_{1} } \right)} }}{{s\sqrt {\left( {s + M} \right)} }}\exp \left( { - y\sqrt {\left( {s + M} \right)} } \right) \\ & \quad - B_{2} \frac{{\sqrt {\Pr \left( {s + \eta_{1} } \right)} }}{{(s + b)\sqrt {\left( {s + M} \right)} }}\exp \left( { - y\sqrt {\left( {s + M} \right)} } \right) - B_{3} \frac{{\sqrt {\Pr \left( {s + \eta_{1} } \right)} }}{{(s + g_{1} )\sqrt {\left( {s + M} \right)} }}\exp \left( { - y\sqrt {\left( {s + M} \right)} } \right) \\ & \quad - B_{4} \frac{{\sqrt {Sc\left( {s + \eta_2} \right)} }}{{s\sqrt {\left( {s + M} \right)} }}\exp \left( { - y\sqrt {\left( {s + M} \right)} } \right) + B_{4} \frac{{\sqrt {Sc\left( {s + \eta_2} \right)} }}{{(s + g_{2} )\sqrt {\left( {s + M} \right)} }}\exp \left( { - y\sqrt {\left( {s + M} \right)} } \right) \\ & \quad + B_{1} \frac{1}{s}\exp \left( { - y\sqrt {\Pr \left( {s + \eta_{1} } \right)} } \right) + B_{2} \frac{1}{s + b}\left( { - y\sqrt {\Pr \left( {s + \eta_{1} } \right)} } \right) + B_{3} \frac{1}{{s + g_{1} }}\exp \left( { - y\sqrt {\Pr \left( {s + \eta_{1} } \right)} } \right) \\ & \quad + B_{4} \frac{1}{s}\exp \left( { - y\sqrt {Sc\left( {s + \eta_2} \right)} } \right) - B_{4} \frac{1}{{s + g_{2} }}\exp \left( {y\sqrt {Sc\left( {s + \eta_2} \right)} } \right) \\ \overline{u} (y,0) & = 0\& \overline{u} (0,s) = f(s), \\ \end{aligned}$$

where

$$\begin{aligned} g_{1} & = \frac{{\eta_{1} {\text{Pr}} - {\text{M}}}}{{{\text{Pr}} - 1}},g_{2} = \frac{{\eta_{2} {\text{Sc}} - M}}{{{\text{Sc}} - 1}},B_{1} = \frac{{ - {\text{Gr}}{.}}}{{g_{1} ({\text{Pr}} - 1)}}, \\ B_{2} & = \frac{{{\text{Gr}}.a}}{{\left( {g_{1} - b} \right)(\Pr - 1)}},B_{3} = \frac{{ - {\text{Gr}}}}{(\Pr - 1)}\left[ {\frac{1}{{g_{1} }} - \frac{a}{{\left( {g_{1} - b} \right)}}} \right],B_{4} = \frac{{ - {\text{Gm}}}}{{g_{2} ({\text{Sc}} - 1)}}. \\ \end{aligned}$$

To obtain the exact analytical solution adopting the inverse Laplace transform of the above equation by using theEqs. (18–23) which is given as under

$$\begin{aligned} u(y,t) & = [( - f(t)) - B_{1} \Pi_{1} (y,t,\eta_{1} ) - B_{2} \Pi_{2} (y,t,\eta_{1} ,b) - B_{3} \Pi_{2} (y,t,\eta_{1} ,g_{1} ) - B_{4} \Pi_{1} (y,t,\eta_{2} ) \\ & \quad + B_{4} \Pi_{2} (y,t,\eta_{2} ,g_{2} )]*\Omega (y,t,M) + B_{1} \Theta (y,t,\Pr ,\eta_{1} ) + B_{2} \Psi (y,t,\Pr ,\eta_{1} ,b) + B_{3} \Psi (y,t,\Pr ,g_{1} ) \\ & \quad + B_{4} \Theta (y,t,Sc,\eta_{2} ) - B_{4} \Psi (y,t,Sc,\eta_{2} ,g_{2} ). \\ \end{aligned}$$

## Special cases

(i) In the absence of heat source ($\eta_{1} = 0$)

In Eq. (8), when we put $\eta_{1} = 0$, then we obtain the solution in the form as given under:

16 $$T\left( {y,t} \right) = erfc\left( {\frac{{y\sqrt {{\text{pr}}} }}{{2\sqrt {\text{t}} }}} \right) - \frac{{ay\sqrt {{\text{pr}}} }}{2\sqrt \pi }e^{ - bt} \int\limits_{0}^{t} {\frac{1}{h\sqrt h }} \exp \left( {\frac{{ - y^{2} {\text{pr}}}}{4h} + {\text{b}}h} \right)dh.$$

The result is uniform to that in the published literature achieved by Rubbab et al.^5^.

(ii) In the absence of mass transfer ($C(y,t) = 0$)

We take $C(y,t) = 0$ and using $\eta_{1} = 0,{\text{ M = 0}}$, in Eq. (8), we achieved the solution after lengthy calculation:

17 $$\begin{aligned} u\left( {y,t} \right) & = \frac{ - \Omega }{{\sqrt \pi }}\int\limits_{0}^{t} {\frac{{f\left( {t - h} \right)}}{\sqrt h }} \exp \left( {\frac{{ - y^{2} }}{4h}} \right)dh \\ & \quad + \frac{{{\text{Gr}}\sqrt {{\text{pr}}} }}{{\text{pr - 1}}}\left[ {\left( {t + \frac{{y^{2} }}{2} - \frac{a}{b}} \right)erfc\left( {\frac{y}{2\sqrt t }} \right) - \frac{y\sqrt t }{{\sqrt \pi }}\exp \left( {\frac{{ - y^{2} }}{4t}} \right) + \frac{{2ae^{ - bt} }}{b\sqrt \pi }\int\limits_{{\frac{y}{2\sqrt t }}}^{\infty } {\exp \left( {\frac{{by^{2} }}{{4h^{2} }} - h^{2} } \right)} dh} \right] \\ & \quad - \frac{{{\text{Gr}}}}{{\text{pr - 1}}}\left[ {\left( {t + \frac{{y^{2} {\text{pr}}}}{2} - \frac{a}{b}} \right)erfc\left( {\frac{{y\sqrt {{\text{pr}}} }}{2\sqrt t }} \right) - \frac{{y\sqrt {{\text{pr}}t} }}{\sqrt \pi }\exp \left( {\frac{{ - y^{2} {\text{pr}}}}{4t}} \right) + \frac{{2ae^{ - bt} }}{b\sqrt \pi }\int\limits_{{\frac{{y\sqrt {{\text{pr}}} }}{2\sqrt t }}}^{\infty } {\exp \left( {\frac{{by^{2} {\text{pr}}}}{{4h^{2} }} - h^{2} } \right)} dh} \right]. \\ \end{aligned}$$

The result is uniform to the one in published literature achieved by Rubbab et al.^5^.

## Graphical results and discussions

**Figure 2:**
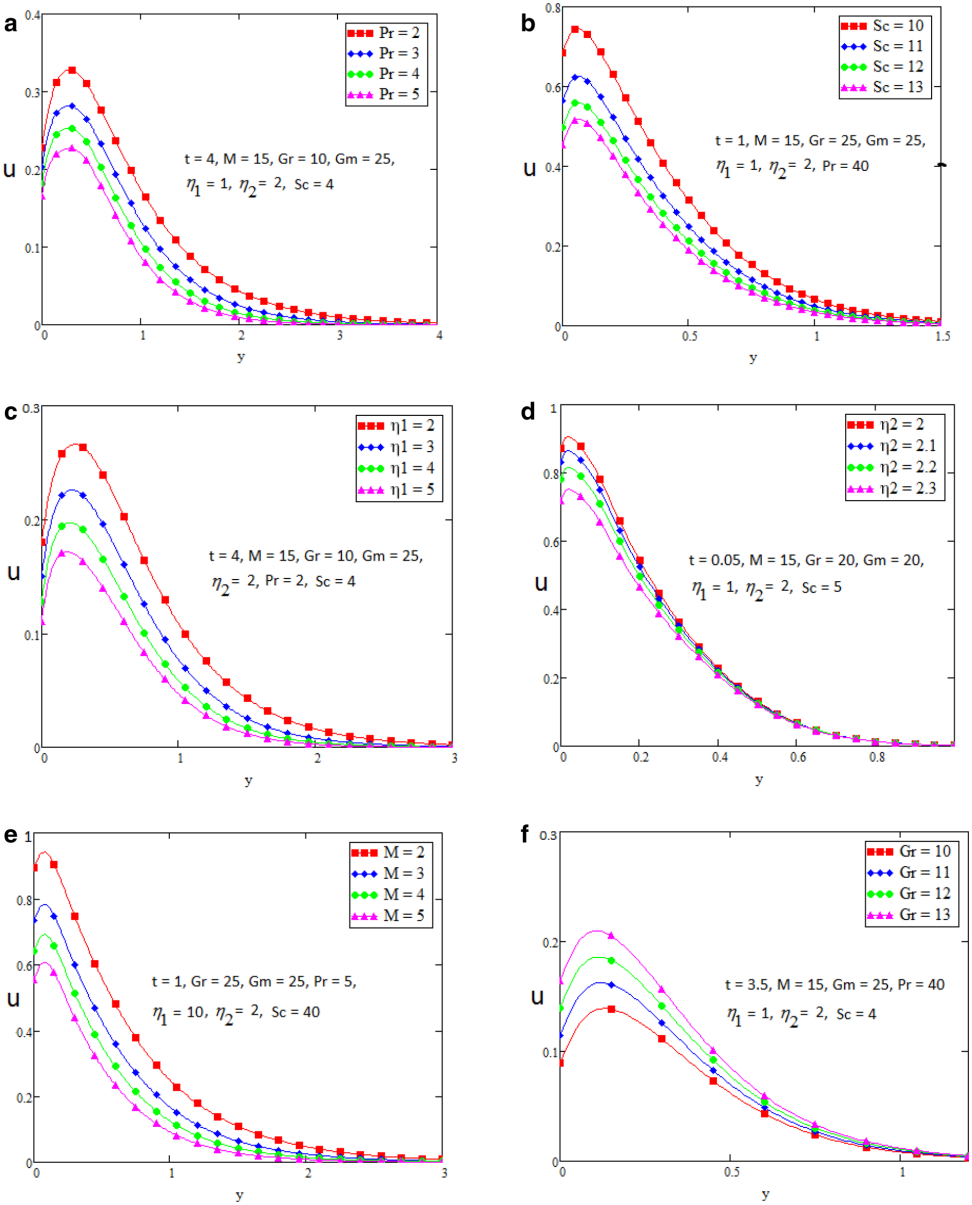
(**a**–**f**) Profiles of the velocities for $f\left( t \right) = \sin wt$.

**Figure 3:**
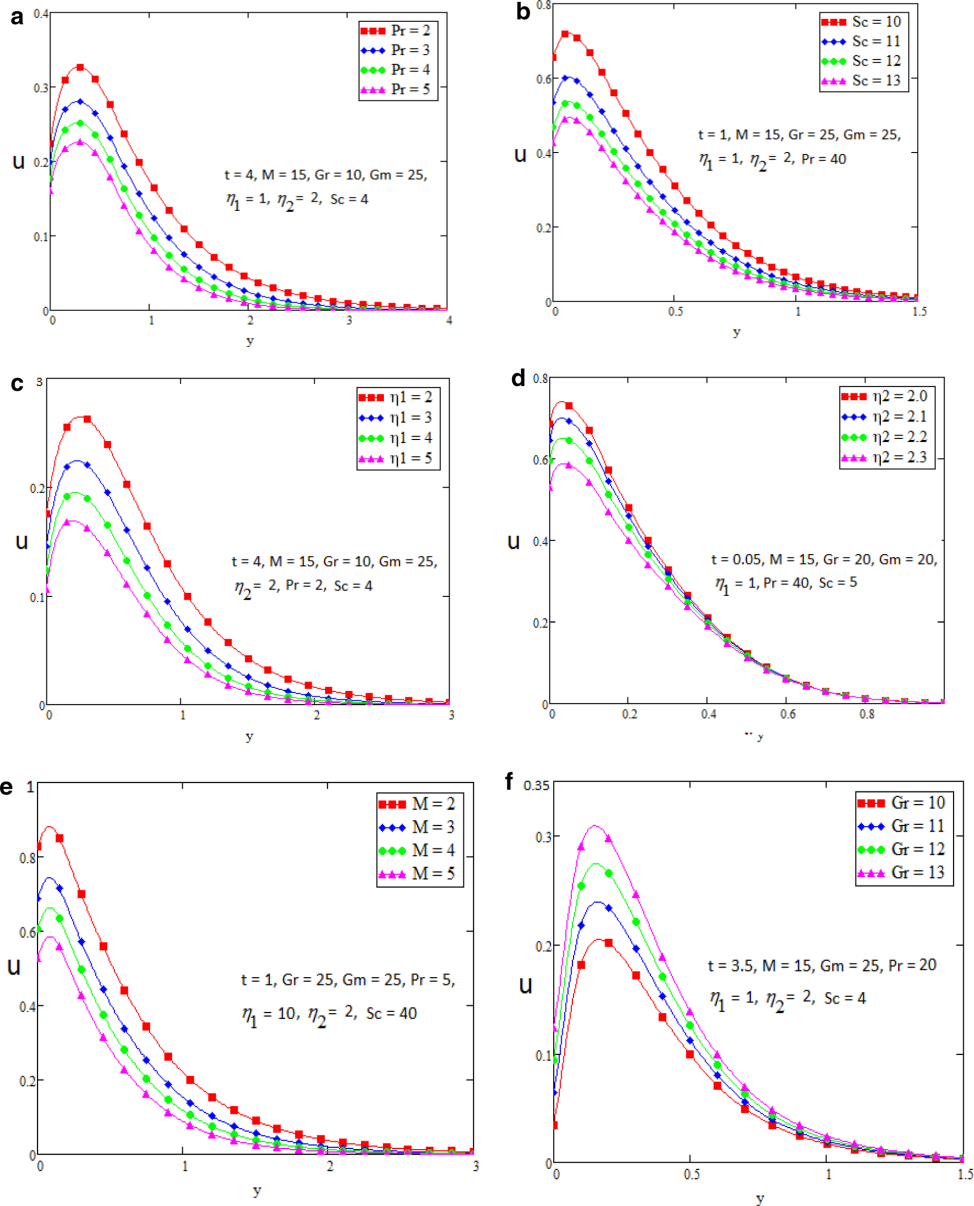
(**a**–**f**) Profiles of the velocities for $f(t) = H(t)$.

**Figure 4:**
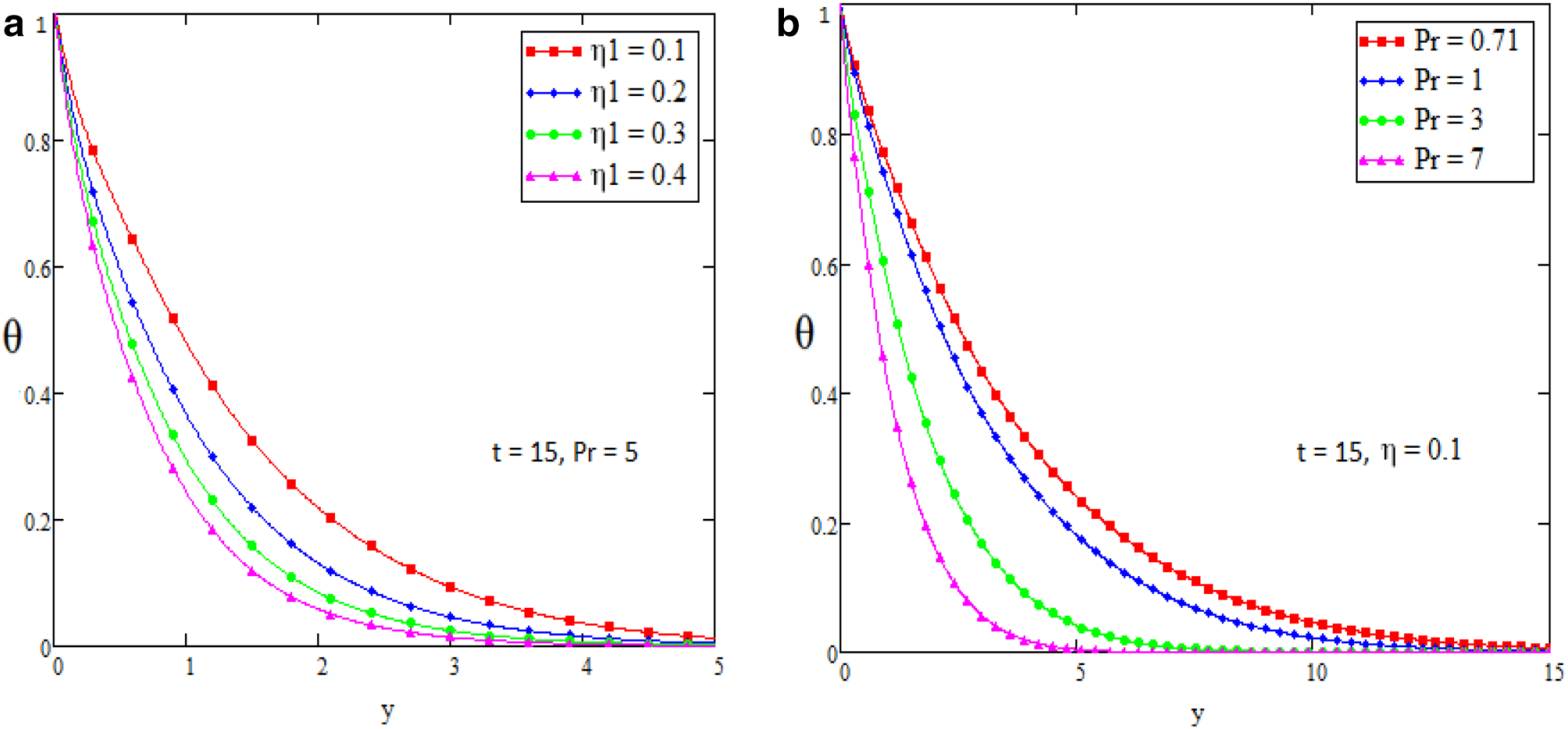
(**a**,**b**) Profiles of the temperatures for distinct values of $\eta_{1}$ and Pr.

**Figure 5:**
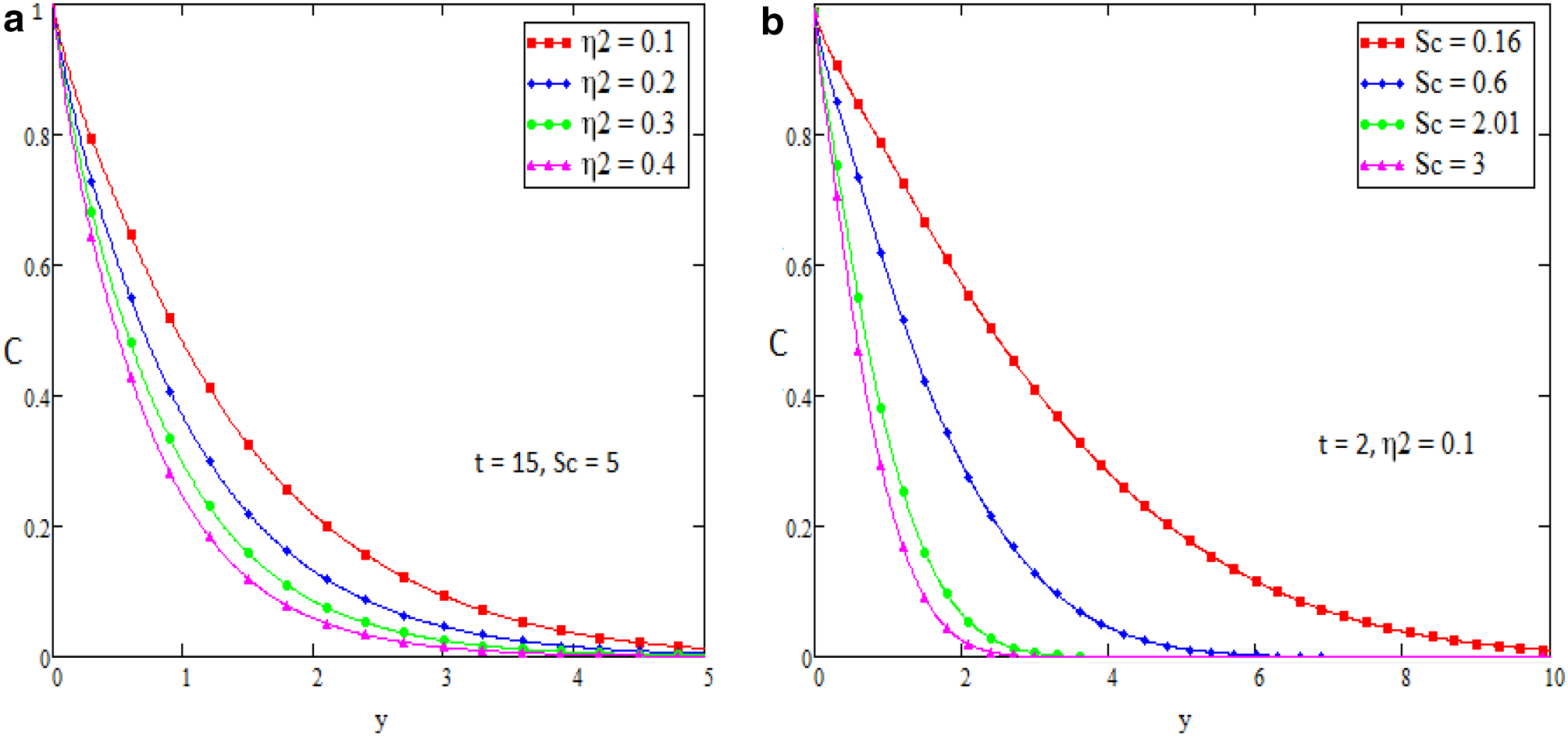
(**a**,**b**) Profiles of the concentration for different values of $\eta_{1}$ and Sc.

**Figure 6:**
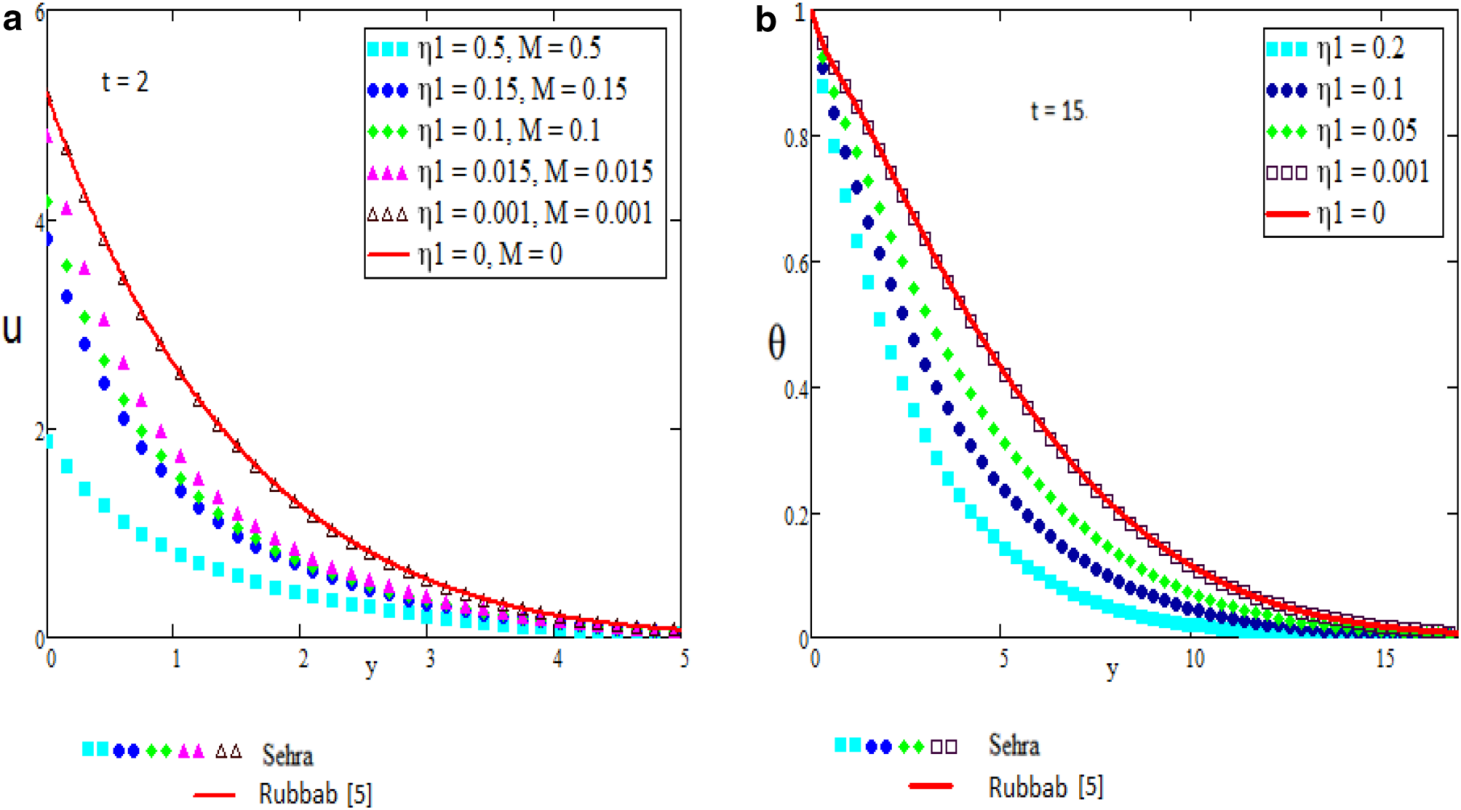
(**a**,**b**) Profiles of the velocity and temperature in comparison with Rubbab^5^.

In this section we present the graphical interpretation and numerical computations in order to receive a clear image of the model equations, the numerical computations performed and the influences of various physical parameters like Prandtl ($\Pr$) number, Schmidt (Sc) number, heat source ($\eta_{1}$), chemical reaction parameter ($\eta_{2}$), MHD parameter (*M*) and Grashof number of thermal (Gr) on flow quantities. Physical diagram of the problem is shown in Fig. 1. The layout of velocity for $f\left( t \right) = \sin wt$ is presented in the Fig. 2a–f. The influence of $\Pr$ on the velocity field is shown in Fig. 2a. It is certain in Fig. 2a that the velocity of the fluid is decreasing for greater values of $\Pr$. After all for higher value of Pr fluids will have higher viscosities and therefore the fluid velocity decrease. Fig. 2b presents the effects of Sc on the fluid velocity. The fluid velocity decelerates for increasing values of Sc. Therefore the motion of the fluid is decreased. The velocity profile for distinct values of $\eta_{1}$ is presented in Fig. 2c, which shows that velocity decrease as we increase the heat source. The influence of $\eta_{2}$ parameter (chemical reaction) on the velocity of the fluid is shown in Fig. 2d. This is noticed that the velocity decelerates with accelerating values of $\eta_{2}$ which represent the chemical reaction. Also the magnetic parameter M effects investigated in Fig. 2e. It is acknowledged that, the supporting effect of MHD on the fluid velocity with electrically conducting fluid, in this process a resistive force arises. It could be noticed that from Fig. 2e. The effect of magnetic parameter M on fluid velocity shows that the velocity decrease when M increase. The consequences of Gr number on fluid velocity is given in Fig. 2f. It is noticed that the fluid velocity decrease, if we decrease the value of Grashof number Gm and velocity increase as the Gm number increases. The thermal Grashof is the ratio of viscous force and thermal buoyancy, which causes free convection. The layout of velocity for $f\left( t \right) = H\left( t \right)$ is presented in the Fig. 3a–f. The analysis in Fig. 3a–f for the motion of fluid is same as in Fig. 2a–f. The layout of temperature profiles is presented in the Fig. 4a,b. Figure 4a,b represent the changes of temperature profile for distinct values of $\eta_{1}$ and $\Pr$. It is acknowledged that heat transfer decreases if we increase the values of $\eta_{1}$ and $\Pr$. The layout of concentration profiles is presented in the Fig. 5a,b. Figure 5a,b shows the mass concentration profile fluctuations for distinct values of $\eta_{2}$ and Sc. It is analyzed from these plots that decreasing the values of $\eta_{2}$ and Sc, concentration profile also decreases. In Fig. 6a,b we compared the obtained solutions as limiting cases with those obtained by Rubbab et al.^5^. For temperature layout the Prandtl consequences are investigated and it is observed that the temperature become higher as we neglect the heat source parameter in our problem and achieved the result published in literature^5^. Grashof influences are checked for velocity profile and it is observed that in the absence of MHD, heat source and chemical reaction the fluid flow rate become higher comparable to our actual problem also in this case we achieved the result published in the literature^5^.

Case 1: when $f\left( t \right) = \sin wt$ then velocity profile shape is in the given figures.

Case 1: when $f\left( t \right) = H(t)$ then velocity profile shape is in the given figures.

Where $H(t)$ is unite step function.

## Conclusions

The study considered here presents the analysis of the unsteady free convective fluid flow of a viscous incompressible fluid in the existence of MHD and the chemical molecular diffusivity effects upon a perpendicular plate with arbitrary time dependent shear stresses and exponential heating phenomena. Special cases are investigated of the obtained solutions and it is noticed that some well-known results are achieved published in literature from these special cases. The profiles (concentration, temperature and velocity) are analyzed graphically for distinct physical parameters. It is observed that.

- Higher value of $\Pr$ number, $\eta_{1}$ parameter, Sc number, $\eta_{2}$ parameter and MHD parameter *M* reduce the motion of fluid, while as in the absence of $\eta_{1}$ parameter, $\eta_{2}$ parameter and MHD parameter *M* the motion of fluid is increasing and achieved the result published in the literature^5^.
- Motion of fluid is increasing for larger values of Gr.
- Temperatures profiles decelerated for higher values of $\eta_{1}$ and $\Pr$ number.
- It is observed that the temperature become higher as we neglect the heat source parameter in our problem and achieved the result published in literature^5^.
- Concentration profile of mass come down with raising points of the $\eta_{2}$, Sc.

## Appendices

18 $$\Phi (y,t,l) = L^{ - 1} \exp ( - y\sqrt {\left( {s + l} \right)} ) = \frac{y}{{2t\sqrt {\pi t} }}\exp \left( { - \left( {lt + \frac{{y^{2} }}{4t}} \right)} \right),$$

19 $$\begin{aligned}\Theta (y,t,l,m) & = L^{{ - 1}} \left( {\frac{1}{s}\exp \left( { - y\sqrt {m\left( {s + l} \right)} } \right)} \right) \\& = \frac{1}{2}\left[ {erfc\left( {\frac{y}{2}\sqrt {\frac{m}{t}} + \sqrt {lt} } \right)e^{{y\sqrt {lm} }} + erfc\left( {\frac{y}{2}\sqrt {\frac{m}{t}} - \sqrt {lt} } \right)e^{{ - y\sqrt {lm} }} } \right],\end{aligned}$$

20 $$\begin{aligned} \Psi (y,t,l,m,n) & = L^{ - 1} \left( {\frac{1}{s + m}\exp \left( { - y\sqrt {n\left( {s + l} \right)} } \right)} \right) \\ & = \frac{1}{2}\exp \left( {lt + \sqrt n } \right)\left[ \begin{gathered} ercf\left( {\frac{y}{2\sqrt t } - \sqrt {(l - m)t} } \right)\exp \left( { - y\sqrt {(l - m)} } \right) \hfill \\ + ercf\left( {\frac{y}{2\sqrt t } + \sqrt {(l - m)t} } \right)\exp \left( {y\sqrt {(a - b)} } \right) \hfill \\ \end{gathered} \right], \\ \end{aligned}$$

21 $$\Omega (y,t,M) = L^{ - 1} \left[ {\frac{1}{{\sqrt {\left( {s + M} \right)} }}\exp \left( { - y\sqrt {\left( {s + M} \right)} } \right)} \right] = \frac{1}{{\sqrt {\pi t} }}\exp \left( { - y^{2} /4t - Mt} \right),$$

22 $$\Pi_{1} (y,t,m) = L^{ - 1} \left[ {\frac{{\sqrt {\Pr \left( {s + m} \right)} }}{s}} \right] = \sqrt {\Pr } \left[ {\sqrt m \times erf\sqrt {mt} + \frac{1}{{\sqrt {\pi t} }}\exp \left( { - mt} \right)} \right],$$

23 $$\Pi_{2} (y,t,m,n) = L^{ - 1} \left[ {\frac{{\sqrt {\Pr \left( {s + m} \right)} }}{s + n}} \right] = \sqrt {\Pr } \left[ {\sqrt {m - n} \times erf\sqrt {(m - n)t} + \frac{1}{{\sqrt {\pi t} }}\exp \left( { - \left( {m - n} \right)t} \right)} \right].$$

## Nomenclature

$$C$$: Fluid concentration
$Gm$: Mass Grashof number
$g$: Acceleration due to gravity
M: Magnetic parameter
$Q$: :Heat generation or absorption coefficient
$$Sc$$: Schmidt number
$$T$$: Fluid temperature
$C_{p}$: Specific heat at a constant pressure
$T_{\infty }$: Fluid Temperature far away from the plate
$C_{\infty }$: Fluid concentration far away from the plate
$$\nu$$: Kinematic viscosity
$\eta$: Non-dimensional slip parameter
$$\varphi$$: Inclination parameter
$$\mu$$: Dynamic viscosity
$D$: Mass diffusion coefficient
$Gr$: Thermal Grashof number
$k$: Thermal conductivity of the fluid
$$\Pr$$: Prandtl number
K: Chemical reactions parameter
S: Laplace transforms parameter
$T_{w}$: Temperature at the plate
$B_{0}$: Uniform magnetic field
$C_{w}$: Concentration at the plate
$$t_{0}$$: Characteristics time
$$\rho$$: Fluid density
$$\sigma$$: Electrical conductivity
$\eta_1$: Non-dimensional heat source
$\eta_2$: Non-dimensional chemical reaction
$\gamma$: Volumetric coefficient of mass expansion
$\beta$: Volumetric coefficient of thermal expansion
